# MKP-3 suppresses LPS-induced inflammatory responses in HUVECs via inhibition of p38 MAPK/NF-κB pathway

**DOI:** 10.1080/19768354.2021.1954551

**Published:** 2021-07-14

**Authors:** Banzragchgarav Unenkhuu, Da Bin Kim, Hong Seok Kim

**Affiliations:** a Department of Molecular Medicine, College of Medicine, Inha University, Incheon, Republic of Korea; b Program in Biomedical Science and Engineering, College of Medicine, Inha University, Incheon, Korea

**Keywords:** MKP-3, endothelial cell, inflammation, lipopolysaccharide, endotoxemia

## Abstract

Endothelial cell dysfunction and inflammatory responses play critical roles in the development of atherosclerosis. Recent data on the processes underlying atherogenesis indicate the substantial role of endotoxins (lipopolysaccharides; LPS) of the intestinal microflora in the initiation and progression of atherosclerosis. Mitogen-activated protein (MAP) kinase phosphatase-3 (MKP-3) is a cytoplasmic dual-specificity protein phosphatase that specifically binds to and inactivates MAP kinases in mammalian cells, but its biological function in endothelial cell dysfunction and inflammatory responses remains largely unknown. The aim of the present study was to investigate the role of MKP-3 in endotoxin-induced endothelial inflammation by western blotting, quantitative polymerase chain reaction, and immunofluorescence. The results of our study demonstrated that MKP-3 overexpression markedly inhibited the adhesion of human monocytic THP-1 cells to human umbilical vein endothelial cells (HUVECs) by downregulating the expression of vascular cell adhesion protein 1 (VCAM-1) and pro-inflammatory cytokines. In contrast, MKP-3-encoding gene knockdown by small interfering RNA (siRNA) exacerbated LPS-induced endothelial dysfunction. Additionally, we found that MKP-3 overexpression inhibited LPS-induced p38 MAPK phosphorylation and decreased the nuclear translocation of nuclear factor kappa B (NF-κB) after LPS treatment, suggesting its implication in the LPS/Toll-like receptor 4 (TLR4)/p38/NF-κB pathway. Overall, these observations suggest that MKP-3 plays a protective role in endothelial dysfunction and may be a therapeutic target.

## Introduction

Endothelial dysfunction plays a critical role in the initiation and maintenance of atherosclerosis and may serve as a marker for future risk of cardiovascular diseases (Davignon and Ganz 2004). Multiple factors, including circulating inflammatory cytokines (Kleinbongard et al. 2010), reactive oxygen species (Kalinowski and Malinski 2004), low-density lipoproteins (Mundi et al. 2018), and lipopolysaccharide (LPS) (Wiedermann et al. 1999; Tang et al. 2013) directly and indirectly induce endothelial damage and activation.

LPS, also known as endotoxin, is the key structural component in gram-negative bacterial cell walls and is one of the main inflammatory mediators in atherogenesis (Alexander and Rietschel 2001; Hack and Zeerleder 2001). It induces inflammation by directly activating the endothelial cells and leukocytes and eliciting a series of specific cell responses, including upregulation of the expression of cell adhesion molecules and inflammatory cytokines in endothelial cells (Bierhaus et al. 2000). This increases permeability of the endothelium and prompts recruitment of monocytes/macrophages to induce inflammation (Cohen 2002). Enhanced vascular permeability and increased monocyte adhesion onto endothelial cells may play important roles in the development of atherosclerosis (Singh et al. 2002).

Mitogen-activated protein kinases (MAPKs) are widely expressed in signal transduction pathways that coordinately regulate gene transcription, protein synthesis, and cell cycle (Widmann et al. 1999). The three major MAPK subfamilies, the extracellular signal-regulated kinases 1/2 (ERK1/2), p38, and Jun N-terminal kinases (JNK), play important roles in human diseases, including cardiovascular diseases (Hoefen and Berk 2002; Muslin 2008). Thus, MAPK pathways that control inflammatory activation of endothelial cells are crucial to limiting atherogenesis. MAPK phosphatases (MKPs) are a family of dual-specificity phosphatases (DUSPs) that can recognize and inactivate MAPKs. Endothelial cells express several MKPs, including MKP-3 (Rossig et al. 2002), which is also termed DUSP6 and predominantly localized in the cytoplasm and dephosphorylates not only ERK1/2 (Muda et al. 1996) but also p38 (Zhang et al. 2011) and JNK (Ndong et al. 2014). Although MKP-3 has been shown to attenuate endothelial inflammation (Whetzel et al. 2009; Leng et al. 2018), Hsu et al. (Hsu et al. 2018) recently suggested that MKP-3 promotes inflammation through inducible expression of intercellular adhesion molecule-1 (ICAM-1) in tumor necrosis factor (TNF)-α-stimulated endothelial cells. Thus, the role of MKP-3 in endothelial inflammation remains controversial.

In this study, we characterized the role of MKP-3 in regulating LPS-induced endothelial inflammatory responses. We demonstrated for the first time that MKP-3 downregulates vascular cell adhesion molecule-1 (VCAM-1), which is important for monocyte-endothelial interactions (Gerszten et al. 1998), via p38 inhibition and attenuates nuclear translocation of nuclear factor kappa B (NF-κB) in LPS-stimulated human umbilical vein endothelial cells (HUVECs).

## Materials and methods

### Reagents

LPS was purchased from Sigma-Aldrich (St. Louis, MO). U0126, SB203580, and SP600125 were purchased from Selleck Chemicals (Houston, TX). Calcein AM was obtained from Thermo Fisher Scientific (Waltham, MA). Antibodies against VCAM-1, NF-κB p65, and phospho-p38 MAPK were obtained from Cell Signaling Technology (Danvers, MA). Antibodies against β-actin and MKP-3 were obtained from Santa Cruz Biotechnology (Dallas, TX) and Abcam (Cambridge, UK), respectively.

### Cell culture

HUVECs were cultured in endothelial cell growth medium (containing 2% (v/v) fetal bovine serum), both obtained from PromoCell (Heidelberg, Germany), at 37°C in an atmosphere with 5% (v/v) CO_2_ and 95% humidity.

### Western blot analysis

Cells were rinsed with ice-cold phosphate-buffered saline (PBS) and lysed on ice in radioimmunoprecipitation assay lysis buffer (50 mmol/L Tris-HCl [pH 7.4], 150 mmol/L NaCl, 1% Nonidet P-40, 0.1% sodium dodecyl sulfate, and 0.5% sodium deoxycholate) supplemented with protease and phosphatase inhibitors. Total protein lysates were separated by sodium dodecyl sulfate-polyacrylamide gel electrophoresis and analyzed by western blotting. Bands were detected by chemiluminescence on a ChemiDoc imaging system (Bio-Rad, Hercules, CA). To control for sample loading, the blots were subsequently stripped and re-probed for β-actin.

### Quantitative real-time PCR

Total RNA was isolated from cells using the AccuPrep Universal RNA Extraction Kit (Bioneer, Daejeon, Republic of Korea), and 1 μg was then used for cDNA synthesis with the AccuPower CycleScript RT PreMix (Bioneer). The resulting cDNA was PCR-amplified with the following appropriate forward and reverse primer pairs, respectively: VCAM-1: 5′-GATTCTGTGCCCACAGTAAGGC-3′ and 5′-TGGTCACAGAGCCACCTTCTTG-3′; monocyte chemoattractant protein-1 (MCP-1): 5′-AGAATCACCAGCAGCAAGTGTCC-3′ and 5′-TCCTGAACCCACTTCTGCTTGG-3′; interleukin (IL)-1β: 5′-CCACAGACCTTCCAGGAGAATG-3′ and 5′-GTGCAGTTCAGTGATCGTACAGG-3′; TNF-α, 5′-CTCTTCTGCCTGCTGCACTTTG-3′ and 5′-ATGGGCTACAGGCTTGTCACTC-3′; and 18S rRNA: 5′-AACCCGTTGAACCCCATT-3′ and 5′-CCATCCAATCGGTAGTAGCG-3′. RT-qPCR was performed and analyzed using a CFX Connect Real-Time PCR detection system (Bio-Rad), and the gene expression levels were normalized to that of 18S rRNA as the housekeeping gene.

### Small interfering RNA–mediated gene silencing

MKP-3-encoding gene was knocked down using predesigned small interfering RNA (siRNA) targeted against human MKP-3 (#1044769) and control siRNA purchased from Bioneer. Transfections were performed using Lipofectamine RNAiMAX (Thermo Fisher Scientific) according to the manufacturer’s instructions. After 2 days of transfection, cells were exposed to LPS as indicated. Protein knockdown was confirmed by western blot analysis.

### Transfection by electroporation

To transiently overexpress MKP-3, a DNA fragment containing full-length human MKP-3 coding sequences (bp 1–1,146) was generated by PCR amplification of MKP-3 plasmid (a kind gift from Dr. Tae Jun Park, Ajou University, Suwon, Republic of Korea) using the forward primer 5′-GCTGGATATCATGATAGATACGCTCAGACC-3′ and reverse primer 5′-TCGAGCGGCCGCTCACGTAGATTGCAGAGAGT-3′, followed by cloning into EcoRV- and NotI-restricted multiple cloning sites of pcDNA3.1 (Invitrogen). DNA electroporation was performed using the Neon Transfection System MPK5000 (Thermo Fisher Scientific). Subsequently, 5 × 10^5^ cells were used per electroporation using Neon Transfection System 100 μL Kit (Thermo Fisher Scientific) under the following condition: one pulse of 1,350 V for 30 ms.

### Endothelial cell-monocyte adhesion assay

The HUVEC monolayer was seeded in a 6-well plate. Subsequently, the cells were stimulated with LPS (1 µg/mL) for 24 h. Human monocytic THP-1 cells were labeled with 5 µM Calcein AM for 30 min in RPMI-1640 medium and were co-cultured with the HUVECs for another 1 h. Then, non-adherent THP-1 cells were removed by gently washing twice using PBS. The adherent THP-1 cells were visualized using a fluorescence microscope (EVOS FL Cell Imaging System, Thermo Fisher Scientific).

### Immunofluorescence analysis of NF-κB p65 nuclear translocation

HUVECs were grown on coverslips and treated with LPS (1 µg/mL) for 30 min. The cells were then fixed with 4% paraformaldehyde in PBS for 15 min and permeabilized with 0.1% Triton X-100. Nonspecific binding was blocked with 5% normal goat serum for 1 h. Then, the cells were incubated overnight with NF-κB p65 antibody at 4°C. Alexa Fluor 594-conjugated goat anti-rabbit IgG (Thermo Fisher Scientific) was used as the secondary antibody for 1 h. Nuclei were stained with Hoechst 33258. The locations of NF-κB p65 were determined using a fluorescence microscope (EVOS FL Cell Imaging System). Results are expressed as the percentage of NF-κB p65-positive cells in the total cells counted from four randomly chosen high-power (×20) fields in each well.

### Statistics

Data were analyzed using SigmaPlot software version 12.5 for Windows (Systat Software Inc., San Jose, CA), which was tested for use in parametric or nonparametric post hoc analysis. Multiple comparisons were performed using the least significant difference method. All data are presented as the mean ± standard error of the mean (SEM) at least three independent experiments. Statistical comparisons of the results were made using analysis of one-way analysis of variance (ANOVA). *P*-values < 0.05 were considered significant.

## Results

### MKP-3 regulates monocyte adhesion to LPS-stimulated HUVECs

**Figure 1.**
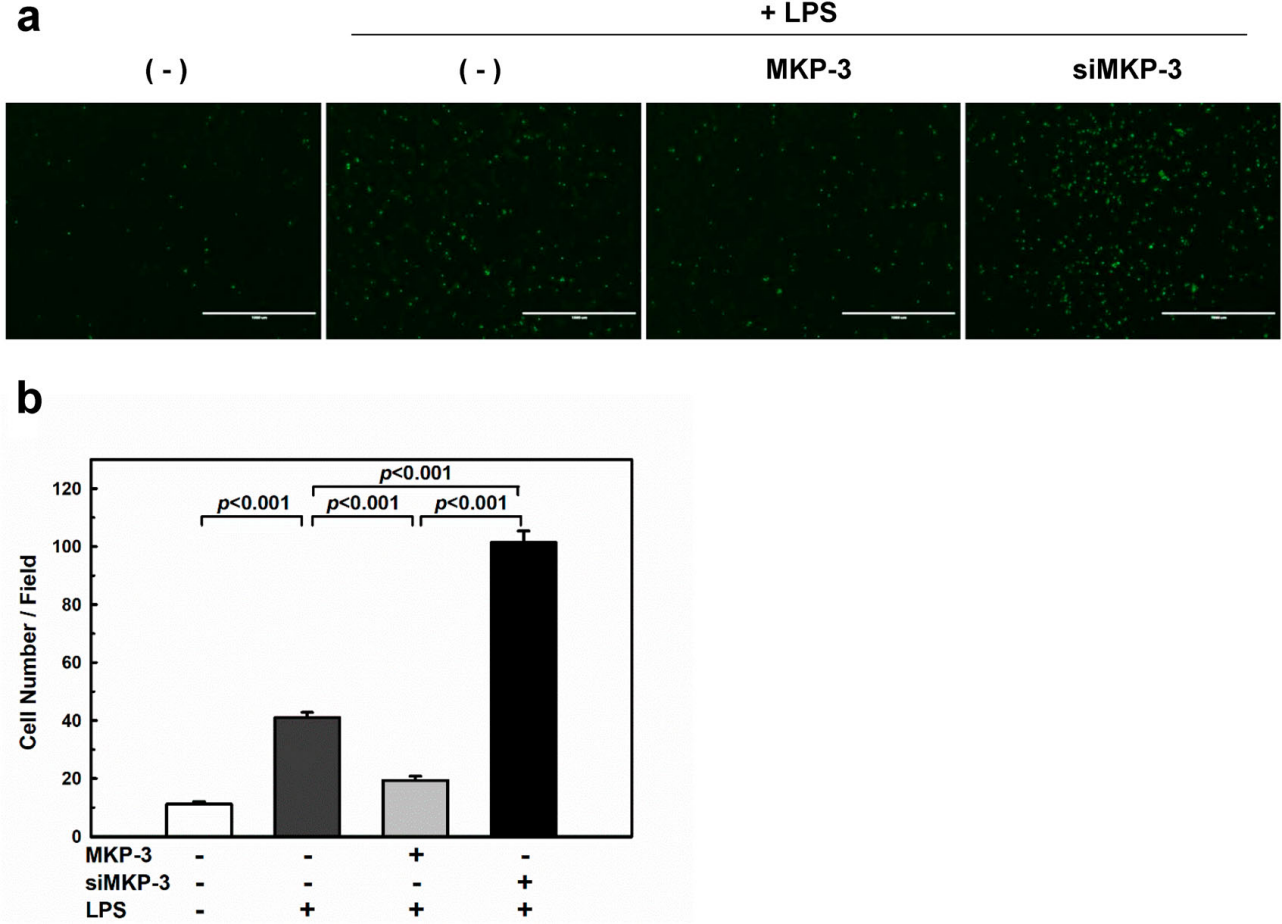
Mitogen-activated protein kinase phosphatase 3 (MKP-3) regulates monocyte adhesion to lipopolysaccharide (LPS)-stimulated endothelial cells. Human umbilical vein endothelial cells (HUVECs) were transfected with either MKP-3 plasmid or MKP-3 siRNA and then treated with LPS (1 µg/mL) for 24 h. Images of the adherent fluorescent THP-1 monocytes were captured using a fluorescence microscope (a) and analyzed (b). Scale bars: 1000 μm. Results shown are the mean ± SEM of three independent experiments.

To examine whether MKP-3 affects endotoxin-induced monocyte-endothelial interaction, HUVECs were transfected with either MKP-3 plasmid or MKP-3 siRNA and then treated with LPS (1 µg/mL) for 24 h. As shown in Figure 1, overexpression of MKP-3 significantly reduced LPS-mediated monocyte adhesion by 52%, whereas knockdown of MKP-3 resulted in a marked increase in monocyte adhesion by 247% in LPS-stimulated HUVECs. These findings suggest that MKP-3 plays a key role in monocyte-endothelial interaction during endotoxin-induced endothelial inflammation.

### MKP-3 negatively regulates VCAM-1 and inflammatory cytokine expression in LPS-stimulated HUVECs

**Figure 2.**
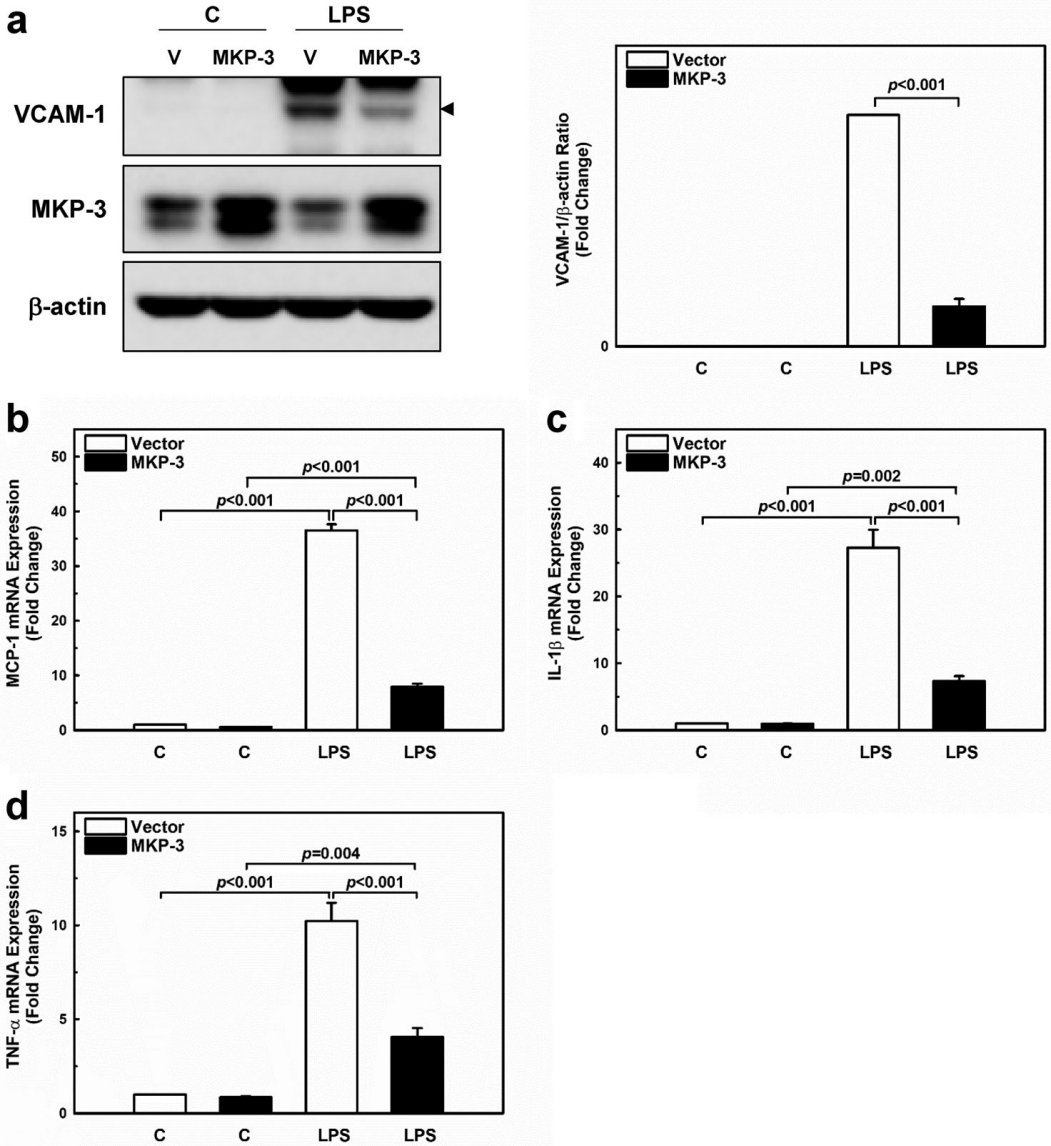
**Overexpression of MKP-3 attenuates LPS-induced inflammatory responses in human umbilical vein endothelial cells (HUVECs).** HUVECs were transfected with either MKP-3 plasmid (MKP-3) or empty vector (V) and then treated with LPS (1 µg/mL) for 24 h. (**a**) Western blot analysis of total cell lysate including VCAM-1 and MKP-3. Arrowhead indicates VCAM-1. (**b-d**) The mRNA levels of inflammatory genes monocyte chemoattractant protein-1 (MCP-1), interleukin (IL)-1β, and tumor necrosis factor (TNF-α) as detected by real-time PCR. 18S rRNA was used as an internal control. The relative mRNA level was normalized to that in the control group of empty vector-transfected HUVECs. Data are mean ± SEM (*n *= 3).

**Figure 3.**
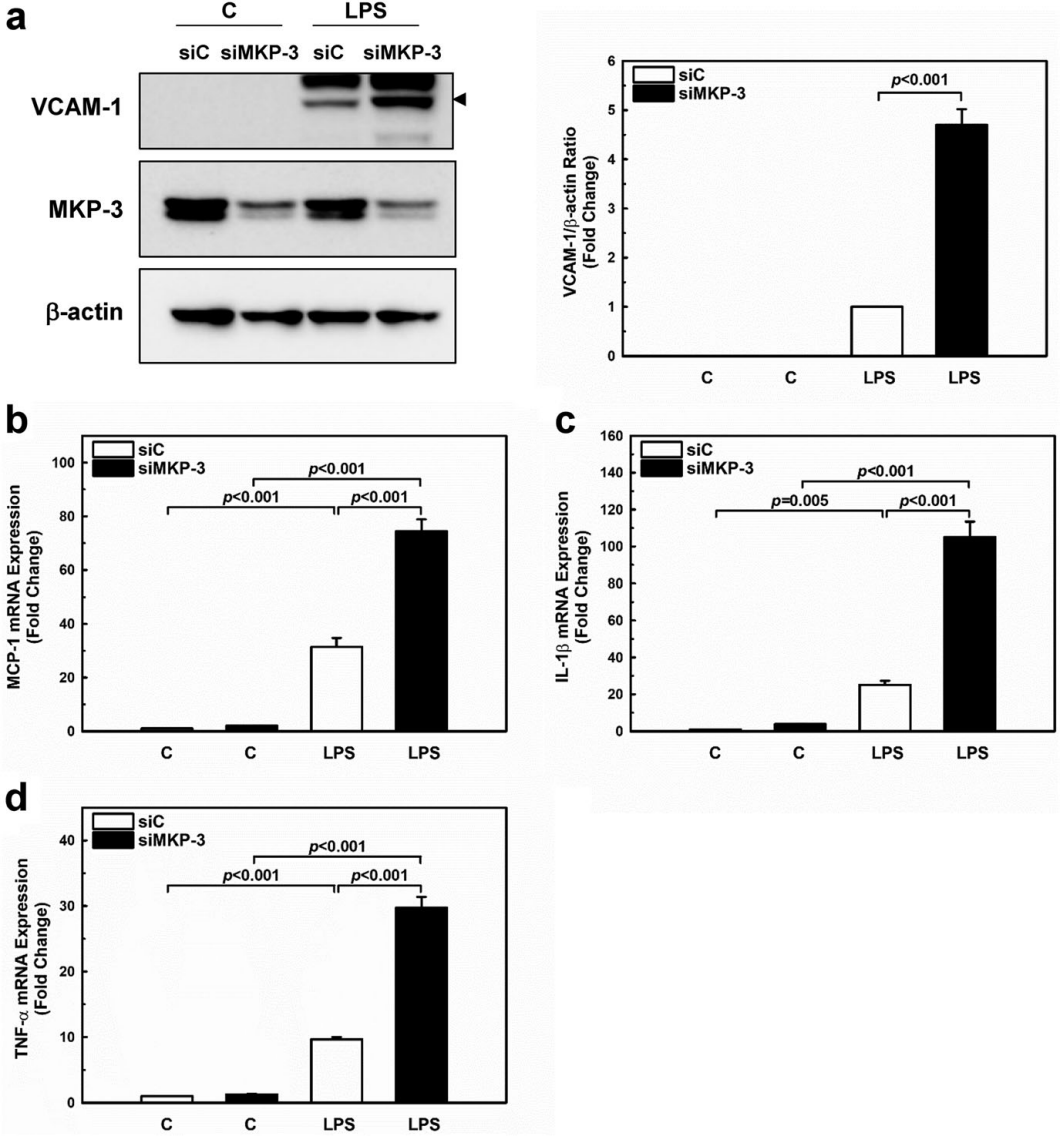
**Knockdown of MKP-3 increases LPS-induced inflammatory responses in HUVECs.** HUVECs were transfected with either MKP-3 specific siRNA (siMKP-3) or control siRNA (siC) and then treated with LPS (1 µg/mL) for 24 h. (**a**) Western blot analysis of protein samples including VCAM-1 and MKP-3. Arrowhead indicates VCAM-1. (**b-d**) The mRNA levels of inflammatory genes monocyte chemoattractant protein-1 (MCP-1), interleukin (IL)-1β, and tumor necrosis factor (TNF)-α as detected by real-time PCR. 18S rRNA was used as an internal control. The relative mRNA level was normalized to that in the control group of control siRNA-transfected HUVECs. Results shown are the mean ± SEM of three independent experiments.

Since endothelial activation in response to LPS results in the induction of VCAM-1, which is an important mediator of mononuclear cell adhesion, cytokines, and chemokines (Mai et al. 2013), we determined whether MKP-3 regulates the expression of pro-inflammatory molecules in LPS-stimulated HUVECs. HUVECs were transfected with MKP-3 and then treated with LPS for 24 h to quantify VCAM-1 and pro-inflammatory cytokine expression. MKP-3 overexpression markedly suppressed VCAM-1 induction in LPS-stimulated HUVECs at protein levels (Figure 2(a)). We then determined whether MKP-3 affects LPS-induced MCP-1, a key chemokine that regulates leukocyte trafficking and modulates leukocyte-endothelial interaction and expression in HUVECs. To this end, we utilized RT-qPCR to analyze MCP-1 mRNA expression. Exposure of HUVECs to LPS increased MCP-1 mRNA expression (36.5-fold), but this effect was remarkably inhibited (7.9-fold) in HUVECs transfected with the MKP-3 plasmids (Figure 2(b)). As shown in Figure 2(c and d), the mRNA expression levels of pro-inflammatory cytokines were also assessed by RT-qPCR. IL-1β and TNF-α were induced by 27.3- and 10.2-fold, respectively. However, these mRNA levels of pro-inflammatory cytokines were significantly reduced upon MKP-3 overexpression. To further ascertain the role of MKP-3 in LPS-induced endothelial inflammation, we knocked down the gene encoding MKP-3 using siRNAs. As shown in Figure 3(a), VCAM-1 protein levels were markedly increased in response to LPS in HUVECs transfected with MKP-3 siRNA. Moreover, these cells also showed significantly higher mRNA expression levels of MCP-1, IL-1β and TNF-α compared to HUVECs transfected with control siRNA (Figure 3(b–d)). These findings suggest that MKP-3 negatively regulates VCAM-1 and inflammatory cytokine induction by LPS in HUVECs.

### MKP-3 suppresses LPS-induced inflammatory responses via the p38 MAPK and NF-κB pathways in HUVECs

**Figure 4.**
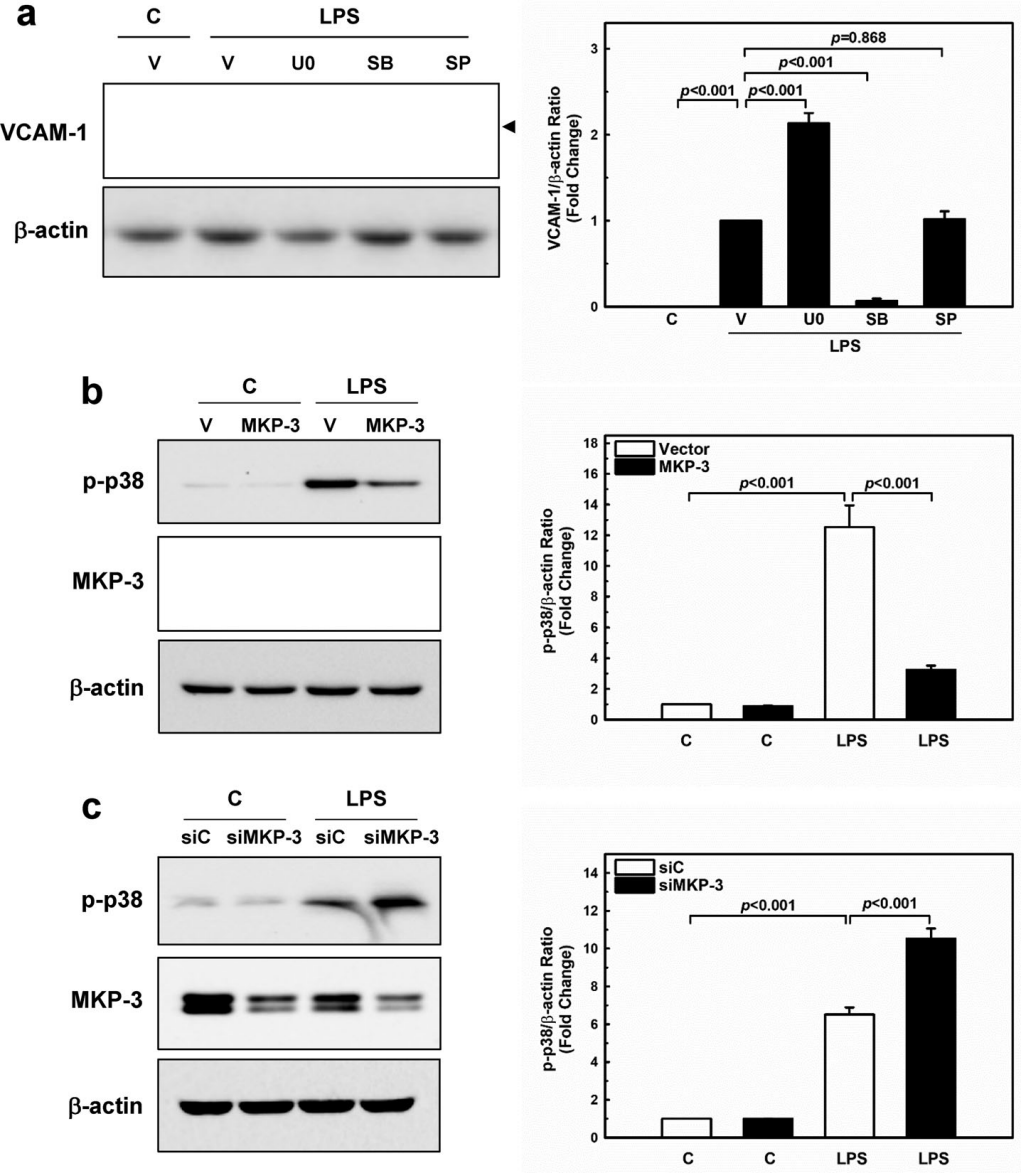
**MKP-3 suppresses LPS-induced inflammatory responses via the p38 MAPK pathway in HUVECs.** (**a**) HUVECs were pre-treated with U0126 (10 µM), SB203580 (10 μM), and SP600125 (10 μM) for 1 h and then stimulated with LPS (1 µg/mL) for 24 h. VCAM-1 levels were assessed by western blot analysis. Arrowhead indicates VCAM-1. (**b**) HUVECs were transfected with either MKP-3 plasmid (MKP-3) or empty vector (V) and then treated with LPS (1 µg/mL) for 30 min. Western blot analysis of total cell lysate including phospho-p38 and MKP-3 levels. (**c**) Westeren blot analysis of HUVECs that were transfected with either MKP-3 specific siRNA (siMKP-3) or control siRNA (siC) and then stimulated with LPS (1 µg/mL) for 30 min. The cells were lysed and analyzed by western blotting with anti-phospho-p38 antibodies and anti-MKP-3 antibodies. HUVECs were transfected with either MKP-3 plasmid or MKP-3 siRNA and then treated with LPS (1 µg/mL) for 30 min.

Stimulation of endothelial cells with LPS can activate several intracellular signaling pathways, such as the MAPKs (Yan et al. 2002; Ahmed et al. 2020) and NF-κB (Ghosh and Hayden 2008). These pathways participate in endothelial inflammation by modulating the expression of adhesion molecules and pro-inflammatory cytokines. To confirm the involvement of the MAPK pathway in the VCAM-1, HUVECs were pre-treated with the MAPK-specific inhibitors U0126 (ERK1/2 inhibitor), SB203580 (p38 inhibitor), and SP600125 (JNK inhibitor) for 1 h, followed by LPS treatment for 24 h. Western blotting results revealed that pre-incubation with SB203580 markedly inhibited VCAM-1 induction, whereas SP600125 had no effect. Notably, ERK1/2 inhibition led to the upregulation of VCAM-1 by LPS (Figure 4(a)). To further investigate whether MKP-3 is necessary for suppression of p38 activation, HUVECs were transfected with either MKP-3 plasmid or MKP-3 siRNA and then stimulated with LPS (1 µg/mL) for 30 min. We observed that the overexpression of MKP-3 significantly inhibited p38 activation (Figure 4(b)). In contrast, silencing of MKP-3 markedly enhanced p38 activation in LPS-stimulated HUVECs (Figure 4(c)), suggesting that MKP-3 plays an essential role in the suppression of p38 activation and VCAM-1 expression.

**Figure 5.**
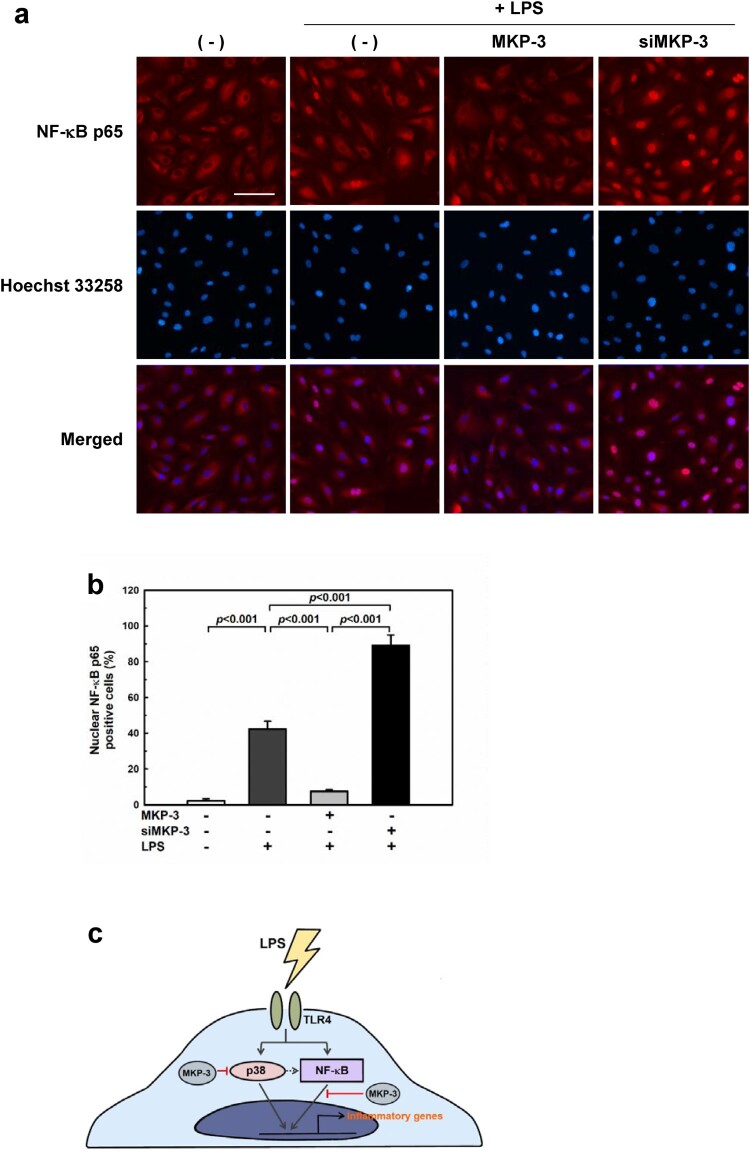
**MKP-3 regulates LPS-induced nuclear translocation of nuclear factor kappa B (NF-κB) in HUVECs.** HUVECs were transfected with either MKP-3 plasmid or MKP-3 siRNA and then treated with LPS (1 µg/mL) for 30 min. (**a**) Representative images of immunofluorescence staining showing NF-κB p65 (red) and nucleus (blue). Scale bars: 100 μm. (**b**) Quantitation of NF-κB p65 nuclear translocation in the indicated groups. Results are shown as the mean ± SEM (*n* = 3). (**c**) A schematic representation of proposed mechanism. MKP-3 may regulate endotoxin-induced endothelial inflammation by inhibiting the activation of p38 and the nuclear translocation of NF-κB.

In addition to p38 MAPK signaling, activation of NF-κB signaling in LPS-induced endothelial cells is well known (Jersmann et al. 2001; Ghosh and Hayden 2008). Since LPS-induced inflammation occurs through TLR signaling and activation of NF-κB, which then translocates to the nucleus to regulate the induced transcription of pro-inflammatory genes (Tak and Firestein 2001), we analyzed the effect of MKP-3 on the cellular distribution of NF-κB. As shown in Figure 5(a and b), immunofluorescence staining of p65 indicated that the NF-κB protein translocated to the nucleus upon LPS treatment. Notably, we found that MKP-3 overexpression abolished the phenotype induced by LPS, resulting in downregulated expression of NF-κB in the nucleus. However, knockdown of the gene encoding MKP-3 promoted the nuclear translocation of NF-κB.

Based on these findings, we propose that MKP-3 can regulate the expression of inflammatory genes by inhibiting p38 activation and decreasing the nuclear translocation of NF-κB (Figure 5(c)).

## Discussion

Atherosclerosis is the main cause of cardiovascular diseases, including myocardial infarction, heart failure, stroke, and claudication. Recent experimental and clinical studies reported that elevated circulating levels of endotoxins are associated with atherosclerosis progression (Lehr et al. 2001; Tang et al. 2013). Even if there is no apparent infection source, high levels of serum endotoxin are observed with metabolic syndromes, such as type II diabetes, obesity, and hypertension (Lassenius et al. 2011). Endothelial cells are the first responder to LPS because they line all blood vessels, (Dauphinee and Karsan 2006) through the activation of pattern recognition receptors (PRRs), accordingly inducing pro-inflammatory cytokines, chemokines, and adhesion molecules (Pober and Sessa 2007). TLR 4 and retinoic acid inducible gene-I (Moser et al. 2016; Lee et al. 2019) are PRRs that lead endothelial activation to LPS. The activation of these receptors by LPS enhances the production of adhesion molecules and pro- inflammatory cytokines through signaling pathways, including the NF-κB and p38 MAPK pathways (Jersmann et al. 2001; Mako et al. 2010). Since p38 plays an important role in inflammatory and stress responses (Hoefen and Berk 2002) and blocking p38 with specific inhibitors results in attenuation of transcriptional activity of NF-κB (Je et al. 2004), MKP-1 and MKP-7 have been suggested to be important regulators of endothelial inflammation (Kiemer et al. 2002; Nizamutdinova et al. 2012).

In the present study, we elucidated that MKP-3 reduces inflammatory response in LPS-stimulated HUVECs. We observed that MKP-3 overexpression inhibited the adhesion of THP-1 monocytes to HUVECs by decreasing the expression levels of VCAM-1 and pro-inflammatory cytokines. Additionally, we found that p38 activation is necessary for VCAM-1 induction and that MKP-3 overexpression markedly abolished p38 activation in LPS-stimulated HUVECs. Although ICAM-1 expression was also induced in HUVECs exposed to LPS (data not shown), we particularly focused on VCAM-1 because VCAM-1 precisely binds to the leukocytes (monocytes and T lymphocytes) found in early human and experimental atheroma (Cybulsky et al. 2001). Importantly, we also revealed that MKP-3 significantly inhibited nuclear translocation of NF-κB by LPS stimulation. The p38 directly or indirectly regulates phosphorylation of NF-κB. Notably, SB203580, a specific inhibitor of p38, had no effect on nuclear translocation of this transcription factor (data not shown), suggesting that p38 regulates NF-κB via multiple independent mechanisms. These observations are supported by an article by Saha et al. (Saha et al. 2007), showing that nuclear translocation and DNA-binding ability of NF-κB is unaffected by p38.

MKP-3 has been suggested to inhibit endothelial inflammation induced by high glucose (Whetzel et al. 2009) and homocysteine (Leng et al. 2018). These studies support our observation that MKP-3 is a negative regulator of endotoxin-induced endothelial inflammation. In contrast, Hsu et al. (Hsu et al. 2018) recently reported that MKP-3 promotes endothelial inflammation through ICAM-1 induction in response to TNF-α stimulation. However, both wild-type and catalytically inactive C293S mutant MKP-3, ectopically expressed in HUVECs, could upregulate the expression of ICAM-1 in this report (Hsu et al. 2018). This might be due to the direct or indirect effects of MKP-3 because TNF-α has been shown to induce intracellular reactive oxygen species, which cause oxidation and inhibition of MKP-3 (Kamata et al. 2005).

In conclusion, to the best of our knowledge, this is the first study to demonstrate the role of MKP-3 in LPS-induced inflammatory responses in HUVECs. A clearer understanding of the functional significance of this phosphatase may provide a potential new therapeutic option to prevent endotoxin-initiated atherogenesis. However, further clinical studies are required to verify whether this hypothesis is valid in patients.
